# ALK Inhibitors or Chemotherapy for Third Line in ALK-positive NSCLC? Real-world Data

**DOI:** 10.1093/oncolo/oyab005

**Published:** 2022-01-28

**Authors:** Mor Moskovitz, Elizabeth Dudnik, Sivan Shamai, Yakir Rotenberg, Noa Popovich-Hadari, Mira Wollner, Alona Zer, Maya Gottfried, Moshe Mishaeli, Shoshana Keren Rosenberg, Amir Onn, Ofer Merimsky, Damien Urban, Nir Peled, Natalie Maimon, Jair Bar

**Affiliations:** Thoracic Cancer Service, Rambam Health Care Campus, Haifa, Israel; Thoracic Cancer Service, Davidoff Cancer Center, Rabin Medical Center, Beilinson Campus, Petah Tikva, Israel; Oncology Division, Tel Aviv Sourasky Medical Center, Affiliated with Sackler Faculty of Medicine, Tel Aviv University, Tel Aviv, Israel; Sharett Oncology Institute, Hadassah Hebrew University Medical Center, Jerusalem, Israel; Department of Oncology, Lin Medical Center, Haifa, Israel; Thoracic Cancer Service, Rambam Health Care Campus, Haifa, Israel; Thoracic Cancer Service, Davidoff Cancer Center, Rabin Medical Center, Beilinson Campus, Petah Tikva, Israel; Department of Oncology, Meir Medical Center, Kfar-Saba, Israel; Department of Oncology, Meir Medical Center, Kfar-Saba, Israel; Department of Oncology, Lin Medical Center, Haifa, Israel; Thoracic Oncology Service, Institute of Oncology, Sheba Medical Center, Tel HaShomer, Ramat Gan, Israel; Oncology Division, Tel Aviv Sourasky Medical Center, Affiliated with Sackler Faculty of Medicine, Tel Aviv University, Tel Aviv, Israel; Thoracic Oncology Service, Institute of Oncology, Sheba Medical Center, Tel HaShomer, Ramat Gan, Israel; Thoracic Cancer Service, Davidoff Cancer Center, Rabin Medical Center, Beilinson Campus, Petah Tikva, Israel; Department of Oncology, Meir Medical Center, Kfar-Saba, Israel; Thoracic Oncology Service, Institute of Oncology, Sheba Medical Center, Tel HaShomer, Ramat Gan, Israel

**Keywords:** ALK rearrangement, ALK inhibitors, targeted therapy, third-line treatment, non-small cell lung cancer

## Abstract

**Objectives:**

ALK inhibitors (ALKi) are the standard-of-care treatment for metastatic ALK-rearranged non-small cell lung cancer (NSCLC) in the first- and second-line setting. We conducted a real-world multi-institutional analysis, aiming to compare the efficacy of third-line ALKi versus chemotherapy in these patients.

**Methods:**

Consecutive ALK-positive metastatic NSCLC patients treated with at least one ALKi were identified in the working databases of 7 Israeli oncology centers (the full cohort). Demographic and clinical data were collected. Patients receiving any systemic treatment beyond 2 ALKi comprised the third-line cohort, whether a third ALKi (group A) or chemotherapy (group B). Groups A and B were compared in terms of overall survival (OS) and time-to-next-treatment line (TNT).

**Results:**

At a median follow-up of 41 months (95% confidence interval [CI]: 32-55), 80 (47.1%) have died. Median OS (mOS) in the full cohort (*n* = 170) was 52 months (95% CI: 32-65). Number of ALKi (hazard ratio [HR] 0.765; 95% CI: 0.61-0.95; *P* = .024) and age (HR 1.02, 95% CI: 1.01-1.04, *P* = .009) significantly associated with OS in the full cohort. The third-line cohort included 40 patients, of which 27 were treated with third ALKi (group A) and 13 treated with chemotherapy (group B). mOS from third-line initiation was 27 months in group A (95% CI: 13-NR) and 13 months for group B (95% CI: 3-NR); the difference was not significant (NS; *P* = .12). Chemotherapy as first line (HR 0.17, 95% CI: 0.05-0.52, *P* = .002) and a higher number of ALKi (HR 0.38, 95% CI: 0.20-0.86, *P* = .011) associated significantly with longer OS of the third-line cohort. TNT was 10 months for group A (95% CI: 5-19) and 3 months for group B (95% CI: 0-NR); the difference was NS (*P* = .079).

**Conclusion:**

We report mature real-world data of more than 4-year mOS in ALK-positive patients. The number of ALKi given was associated with a better outcome. OS and TNT demonstrated a statistically nonsignificant trend for a better outcome in patients receiving a third-line ALKi.

## Teaser

ALK inhibitors are the standard-of-care treatment for metastatic ALK-rearranged non-small cell lung cancer in the first- and second-line setting. This article reports a real-world multi-institutional analysis, comparing the efficacy of third-line ALK inhibitors versus chemotherapy.

**Implications for Practice**

In this retrospective real-world cohort of ALK-positive NSCLC, all treated with ALKi (*n* = 170, collected from 7 Israeli cancer institutes), our goal was to assess whether following the failure of 2 ALKi, patients might benefit more from chemotherapy versus an additional ALKi, since no prospective data are available. This cohort demonstrated a strikingly long OS (52 months, with a 47.1% maturity), and the number of ALKi treatment lines correlated with survival, both in univariate as well as multivariate analysis, but not the number of chemotherapy lines and the courses of radiotherapy administered. Among patients treated with third ALKi, we observed a numerically longer survival compared with chemotherapy, although not in a statistically significant manner. Chemotherapy administration as the first line turned out to be a positive prognostic factor, both on univariate as well as on multivariate analysis.

## Introduction

Approximately 5% of patients with non-small cell lung cancer (NSCLC) harbor rearrangement in the anaplastic lymphoma kinase *(ALK)* gene, a potent oncogenic driver,^1,2^ most commonly younger patients, with adenocarcinoma and never smokers.^3^ Tyrosine kinase inhibitors (TKIs) targeting ALK have become the standard of care in the first- and second-line treatment of ALK-rearranged NSCLC patients.^3^ Crizotinib was the first ALK inhibitor (ALKi) introduced, with the high response rate in early-phase trials,^4^ and higher response rate (RR) and progression-free survival (PFS) when compared with chemotherapy in the second- and first-line setting,^5,6^ as well as a trend for better OS.^7^ Resistance to Crizotinib is inevitable, developing within a median of 10-12 months.^8^ Several second-generation ALKi were developed and assessed initially for crizotinib-resistance tumors. Ceritinib, alectinib, lorlatinib, and brigatinib demonstrated high response rate as second-line treatments after crizotinib failure in phase I and II trials.^9-14^ Alectinib and brigatinib improved PFS in the first-line setting when compared with crizotinib.^15-17^ Next-generation agents were mostly tested as a second-line treatment following crizotinib failure, although the current standard of care in the first-line setting is alectinib or brigatinib. The efficacy of second-generation ALK inhibitors for tumors developing resistance to second-generation ALKi was assessed mostly retrospectively,^18^ and data on the efficacy of ALKi in the third-line setting and beyond are scarce. Lorlatinib, an advanced-generation ALK inhibitor, was the only agent whose efficacy as third-line ALKi and beyond was assessed on a prospective, noncomparative trial,^14^ demonstrating a favorable response rate and PFS. As ALK inhibitors demonstrated significant efficacy and favorable toxicity profile, it has become standard of care to offer ALK-positive patients consecutive ALK inhibitors starting from first-line therapy, although the optimal sequence of agents and role of chemotherapy has not been defined. In general, it can be seen that at each additional line of ALKi, its efficacy is attenuated. We questioned the value of a third ALKi following the failure of 2 ALKi treatment lines. We speculated that in such a scenario, patients might be better served by switching to chemotherapy treatment. Therefore, in this study, we retrospectively assessed the real-world impact of a third-line ALKi versus treatment with chemotherapy for ALK-positive advanced NSCLC patients.

## Methods

### Patient Selection and Data Collection

Consecutive patients with ALK-positive (either by fluorescence in situ hybridization, immunohistochemistry using D5F3 antibody, or next-generation sequencing) metastatic NSCLC patients treated with at least one ALKi from January 2012 to January 2020 were identified through internal databases searches of 7 participating Israeli cancer centers/oncology departments (Institute of Oncology, Rambam Medical Center; Davidoff Cancer Center, Rabin Medical Center; Sheba Medical Center, Tel HaShomer; Institute of Oncology, Meir Medical Center; Tel Aviv Sourasky Medical Center; Hadassa Medical Center; and The Clalit Lin Medical Center). These patients constituted the full cohort of patients analyzed in this study. Patients that had received at least 2 lines of ALKi (regardless of previous or intervening non-ALKi treatment lines) and started a next-line treatment (ALKi or non-ALKi) were defined in this analysis as the third-line cohort, and the treatment initiated at that point, either ALKi or non-ALKi (titled from here on as chemotherapy, based on the actually administered treatments in almost all cases), was defined as the third-line treatment of interest. Baseline demographic, clinical, and pathologic characteristics, as well as data on systemic therapy and radiotherapy (XRT) administration, were retrieved from electronic medical records (EMR). XRT courses were defined as definitive or palliative based on the technique and the defined goal of treatment. Stereotactic treatment or concomitant chemoradiotherapy courses were defined as definitive XRT. XRT courses were counted based on the number of treated sites. Response assessments were not collected in this retrospective analysis, nor did we attempt to evaluate PFS.

### Study Endpoints and Statistical Analysis

Study endpoints were OS from diagnosis of advanced disease, OS from initiation of third line of interest for the third-line cohort, and time-to-next-treatment (TNT) for this cohort. Time-to-event analyses were conducted by the Kaplan–Meier method. Overall survival (OS) was calculated from diagnosis of advanced disease till death or censured at last follow-up. For the third-line cohort, OS was calculated also from the initiation of third line of interest till death or censured at last follow-up. TNT was calculated based on treatments recorded in EMR, only for the third-line cohort, from initiation of the third line of interest till initiation of next treatment line, death, or censured at last follow-up if a next treatment line was not initiated. Patients whose third line of interest was an ALKi (group A) were compared with patients getting chemotherapy (group B), in terms of OS and TNT by log-rank test.

Follow-up period was calculated from the diagnosis of advanced disease till the last follow-up or censured at death.

Categorical and ordinal variables were tested for significance by Fisher’s test. Categoric parameters included sex, brain metastasis at diagnosis of advanced disease (presence or absence), first-line treatment for advanced disease (ALKi or chemotherapy), and third line of interest (ALKi or chemotherapy). Ordinal factors examined included the number of definitive XRT courses and the number of palliative XRT courses, categorized into 3 groups (0, 1, or 2 and more treatment courses).

All continuous parameters were tested for normal distribution by Shapiro–Wilk test. Comparisons were done by 2-sided Student’s *t* test. In the event the distribution was found to be significantly different than normal, the Wilcoxon rank-sum test was used. Continuous variables included age, the total number of chemotherapy treatment lines, the total number of ALKi treatment lines, and time from diagnosis of advanced disease to start of third line of interest in months.

All the above parameters were tested for association with OS by cox regression as univariate analysis, followed by multivariate analyses. Multivariate analysis included all parameters demonstrated to have a *P*-value less than .1 on univariate analysis as well as age, sex, and third line of interest (ALKi or chemotherapy). *P* values less than .05 were considered statistically significant.

### Ethics

The study was approved by the local ethics committee at each of the participating centers.

## Results

### Patients and Tumor Characteristics

A total of 170 patients with advanced NSCLC harboring ALK rearrangement, who were treated with at least one line of ALKi were identified at 7 oncologic centers across Israel between January 2012 and January 2020. The demographic and clinical characteristics of the patients are given in Table 1. Of 170 patients, approximately half were men, the median age was 60 (range 20-89 years). Forty-nine patients (28.8%) presented with brain metastases at diagnosis, and 38 (22.3%) were diagnosed with brain metastases while on ALKi. ALK rearrangement was detected using fluorescence in-situ hybridization (FISH) break-apart test in 89 patients, using immunohistochemistry (IHC) in 81 patients and using next-generation sequencing (NGS) in 17 patients. For 25 patients, both IHC and FISH were carried out, of these in 11 cases discordance was found between the tests (in 4 cases IHC positive, FISH negative; in 7 IHC negative, FISH positive). Only in one of these discordant cases a third test was carried out (IHC negative, FISH positive, NGS negative). In 9 cases, NGS was done as well as another test (IHC or FISH). In 4 of these 9 cases, discordances were seen (NGS+, FISH-, IHC-; NGS-, FISH+, IHC-; NGS+FISH-; NGS-, IHC+).

**Table 1.** Clinical and demographic characteristics of metastatic ALK-positive non-small cell lung cancer patients.

	Full cohort *N* = 170	Third-line cohort (*n* = 40)		
Parameters:		Group A *N* = 27	Group B *N* = 13	*P*-value^a^
Men, *n* (%)	84 (49.4)	16 (59.3)	6 (46.2)	.509
Age, years, median (range)	60 (20-89)	55(20-89)	59 (33-77)	.613
BM at diagnosis of advanced disease, *n* (%)	49 (28.8)	7 (25.9)	2 (18.2)	1.000
Method of ALK testing, *n* (%)				.632
FISH	89 (52)	16 (59)	8 (62)	
IHC	81 (48)	11 (41)	3 (23)	
NGS	17 (10)	2 (7)	0 (0)	
Missing data	17 (10)	3 (11)	2 (15)	
Treatment, *n* (%)				
**Chemotherapy Tx lines, median (range)** **[Average]**	**0 (0-4)**	**1 (0-4)** **[0.78]**	**1 (1-2)** **[1.23]**	**.011**
**ALKi Tx lines - median (range)** **[Average]**	**1 (1-5)**	**3 (3-5)** **[3.44]**	**3 (2-4)** **[2.62]**	**<.001**
Received chemotherapy before first ALKi	44 (25.9)	11 (40.7)	3 (23.1)	.316
Time from diagnosis of advanced disease to start of third line Tx, months, median (range)		23(6-66)	21(8-51)	.885
XRT, number of course (%)				
Definitive				.551
0	101 (59.4)	12 (44.4)	7 (53.8)	
1	43 (25.3)	11 (40.7)	3 (23.1)	
≥2	26 (15.3)	4 (14.8)	3 (23.1)	
Palliative				.879
0	115 (67.6)	15 (55.6)	9 (69.2)	
1	41 (24.1)	9 (33.3)	3 (23.1)	
≥2	14 (8.2)	3 (11.1)	1 (7.7)	
First-line ALKi-N^b^	170 (100)			
Crizotinib	121 (71.2)	24 (88.9)	11 (84.6)	
Ceritinib	1 (0.6)			
Alectinib	45 (26.5)	3 (11.1)	2 (15.4)	
Brigatinib	1 (0.6)			
Ensartinib	1 (0.6)			
Lorlatinib	1 (0.6)			
Second-line ALKi-N^b^	82 (48.2)			
Crizotinib	3 (1.8)			
Certinib	33 (19.4)	16 (59.3)	5 (38.5)	
Alectinib	35 (20.6)	9 (33.3)	6 (46.2)	
Brigatinib	11 (6.5)	2 (7.4)	2 (15.4)	
Third-line ALKi-N^b^	34 (20)			
Crizotinib	1 (0.6)	1 (3.7)		
Alectinib	18 (10.6)	15 (55.6)	3 (23.1)	
Brigatinib	12 (7.1)	9 (33.3)	3 (23.1)	
Lorlatinib	3 (1.8)	2 (7.4)	1 (7.7)	

Parameters that differ in a statistically significant manner between groups A and B are in bold.

Percentages in each case are of the total of the full cohort or out of group A or B, respectively.

*P*-value for the statistical difference between group A and group B;

Treatment lines numbering here refer only to lines of ALKi. Note that some of the patients in group B have received a third-line ALKi, later than the third-line of interest as defined above.

Abbreviations: ALK, anaplastic lymphoma kinase; ALKi, ALK inhibitor; FISH, fluorescence in-situ hybridization; IHC, immunohistochemistry; NGS, next-generation sequencing; Tx, treatment; XRT, radiotherapy.

Patients were treated with a median of 2 lines of treatment (range 1-8), including a median of one ALKi (range 1-5). Forty-four patients (25.9%) were treated with chemotherapy before receiving any ALKi. In 25 of these 44 patients (57%), the reason for the treatment switch was the receipt of the ALK positivity report, and in 2 (5%), the reason was toxicity.

Sixty-nine patients (40.6%) received at least one course of definitive XRT, 55 (32.3%) received at least one course of palliative XRT. Of the patients with brain metastases, 32 (65%) were treated with stereotactic radiosurgery and 29 (59.1%) treated with whole brain radiotherapy. Crizotinib was the most common first ALKi (71.2% of the full cohort) the next being alectinib (26.5%). Eighty-two patients (48.2%) received a second ALKi line, mostly alectinib (20.6% of the full cohort), or ceritinib (19.4%). Thirty-four patients (20% of the full cohort) got a third ALKi, mostly alectinib (10.6% of the full cohort) and brigatinib (7.1% of the full cohort).

We next focused on the third-line cohort (*n* = 40; 23.5% of the full cohort), patients who received further treatment after 2 ALKi. This group did not differ the full cohort by age, sex, and rate of brain metastases. Of the third-line cohort, 27 patients (67.5% of this cohort) were treated with ALKi immediately following the second ALKi (group A), and 13 patients (32.5%) were treated with other therapy, mostly chemotherapy at this point (group B). The ALKi used in the third-line cohort in group A were most commonly alectinib (55.6% of patients), followed by brigatinib (33.3%), and minority were treated with crizotinib (3.7%) and lorlatinib (7.4%). Treatment regimens for group B included platinum-pemetrexed doublet (8 patients, 62%), pemetrexed alone (3 patients, 23%), vinorelbine alone (one patient, 7.5%), and only one patient treated with pembrolizumab alone (7.5%); this group is referred to as the chemotherapy group. The number of ALKi treatment lines and chemotherapy lines differed as expected between groups A and B (Table 1).

### Overall Survival Analysis

With a median follow-up of 41 months (95% CI: 32-55), 80 (47.1%) of the full cohort (*n* = 170) have died. The median OS of the full cohort from diagnosis of advanced disease was 52 months (95% CI: 32-65; Figure 1). The only factors associated with longer OS of patients in the full cohort were younger age and a higher number of lines of ALKi, both correlating significantly with the better OS on univariate as well as multivariate analysis (Table 2).

**Table 2.** Overall survival from diagnosis of advanced disease of metastatic ALK-positive non-small cell lung cancer patients- cox proportional-hazards model of univariate and multivariate analysis of the full cohort (*n* = 170).

	Univariate analysis		Multivariate analysis	
Parameters	HR (95% CI)	*P*-value	HR (95%CI)	*P* -value
Female vs Male	0.78 (0.50-1.21)	.269	0.69 (0.44-1.09)	.113
**Age**	**1.02 (1.00-1.04)**	**.005**	**1.02 (1.01-1.04)**	**.009**
First-line chemotherapy vs ALKi	0.87 (0.53-1.41)	.574		
BM at diagnosis	0.92 (0.57-1.50)	.756		
Definitive XRT (*N* of courses)				
0	Reference			
1	0.80 (0.48-1.34)	.399		
≥2	0.65 (0.34-1.24)	.194		
Palliative XRT (*N* of courses)				
0	Reference			
1	1.21 (0.73-2.00)	.452		
≥2	1.26 (0.62-2.56)	.529		
Total *N* chemotherapy lines	1.10 (0.88-1.37)	.418		
**Total N ALKi lines**	**0.765 (0.61-0.96)**	**.024**	**0.77 (0.61-0.97)**	**.026**

Statistically significant parameters are highlighted in bold.

Abbreviations: ALK, anaplastic lymphoma kinase; ALKi, ALK inhibitor; BM, brain metastases; CI, confidence interval; HR, hazard ratio; Tx, treatment; XRT, radiotherapy.

**Figure 1.**
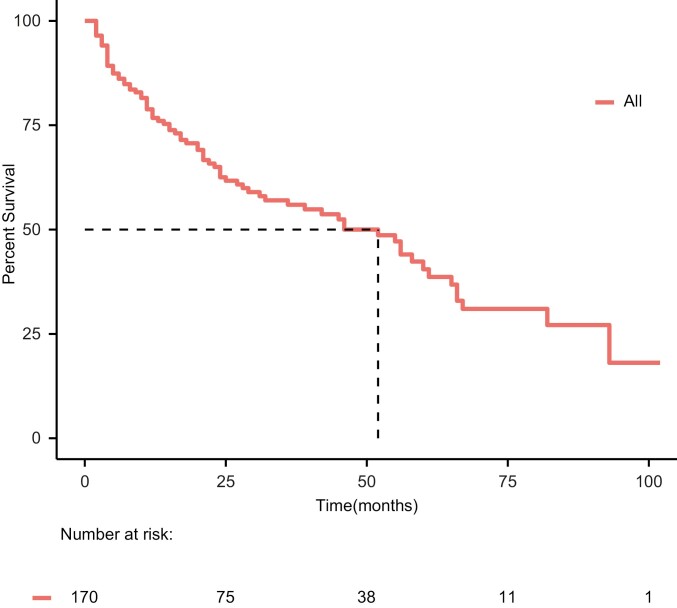
Overall survival of anaplastic lymphoma kinase-positive patients from diagnosis of metastatic disease; the full cohort. Median overall survival = 52 months (95% CI: 32-65).

**Figure 2.**
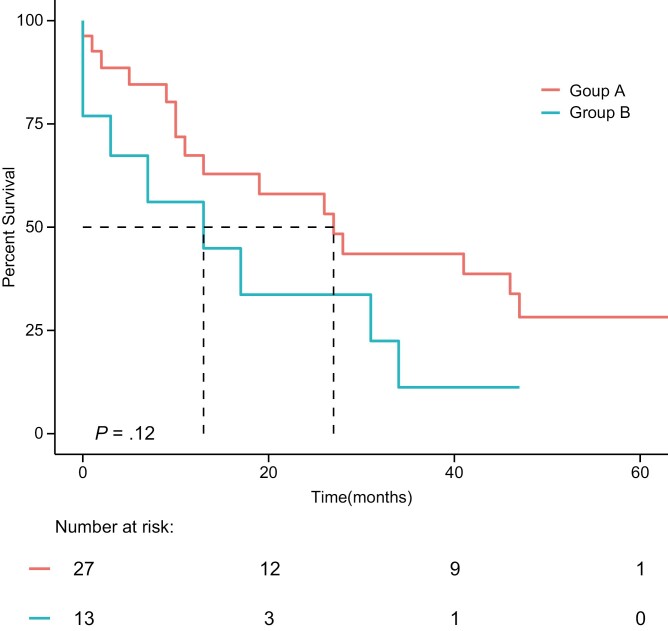
Overall survival from initiation of third-line of interest of anaplastic lymphoma kinase (ALK)-positive patients according to the treatment given as the third line of interest (ie, following 2 ALKi courses); further ALKi (group A) or chemotherapy (group B); third-line cohort (*n* = 40). Group A—median OS 27 months (95% CI: 13-NR). Group B—median OS 13 months (95% CI: 3-NR; *P* = .12).

In the third-line cohort, 25 (62.5%) of the patients have died, including 16 (59.3%) patients in group A, and 9 (69.2%) patients in group B. The median OS from initiation of the third line of interest in the third-line cohort was 27 months (95% CI: 13-NR) in group A, and 13 months (95% CI: 3-NR) in group B (*P* = .12; Figure 2). Regarding OS from diagnosis of advanced disease, OS was 65 months (95% CI: 32-NR) for group A and 55 months for group B (95% CI: 46-NR; *P* = .12; Supplementary Figure S1).

The factors associated with longer OS of patients in the third-line group, when calculated from the initiation of third line of interest, were the administration of chemotherapy treatment as first-line therapy and a larger number of ALKi treatment lines. Both of these factors were significant on univariate as well as on multivariate analysis (Table 3). Third line of interest treatment with ALKi or chemotherapy was not associated with improved OS in a statistically significant manner neither on univariate nor on multivariate analysis. As a sensitivity analysis, we analyzed the factors correlating with the survival of these 2 groups when calculated from diagnosis of advanced disease. On univariate as well as multivariate analysis, only first-line treatment with chemotherapy was associated with better OS (Supplementary Table S1). The difference in survival between groups A and B was not statistically significant nor by hazard ratio (HR) evaluation of the Kaplan–Meier survival curves nor by evaluation of chance of survival at landmark time points (data not shown).

**Table 3.** Cox proportional-hazards model of univariate and multivariate analysis of overall survival from start of third line of interest for ALK-positive patients treated with 2 ALKi and beyond; third-line cohort (*n* = 40).

	Univariate analysis		Multivariate analysis	
Parameters	HR (CI 95%)	*P*-value	HR (CI 95%)	*P*-value
Female vs Male	0.89 (0.40-1.97)	.777	0.59 (0.23-1.53)	.282
Age	1.01 (0.98-1.04)	.533	1.01 (0.98-1.05)	.422
First-line chemotherapy vs ALKi	**0.26 (0.10-0.68)**	**.004**	**0.17 (0.05-0.52)**	**.002**
BM at diagnosis	1.05 (0.41-2.65)	.921		
Definitive XRT (*N* of courses)				
0	Reference			
1	0.69 (0.28-1.70)	.417		
≥2	0.60 (0.20-1.78)	.361		
Palliative XRT (*N* of courses)				
0	Reference			
1	0.75 (0.29-1.95)	.560		
≥2	1.35 (0.43-4.13)	.602		
Total *N* chemotherapy lines	0.94 (0.55-1.59)	.813		
**Total *N* ALKi lines**	**0.41 (0.20-0.86)**	**.018**	**0.38 (0.20-0.86)**	**.011**
Time from diagnosis of advanced disease to start of third-line Tx	0.99 (0.97-1.03)	.929		
Group A vs Group B	0.52 (0.23-1.18)	.119	1.15 (0.37-3.57)	.803

Statistically significant factors are highlighted in bold.

Group A; further ALKi immediately following 2 ALKi, group B; chemotherapy immediately following 2 ALKi.

Abbreviations: ALK, anaplastic lymphoma kinase; ALKi, ALK inhibitor; BM, brain metastases; CI, confidence interval; HR, hazard ratio; Tx, treatment; XRT, radiotherapy.

### Time-to-next-treatment Analysis

**Figure 3.**
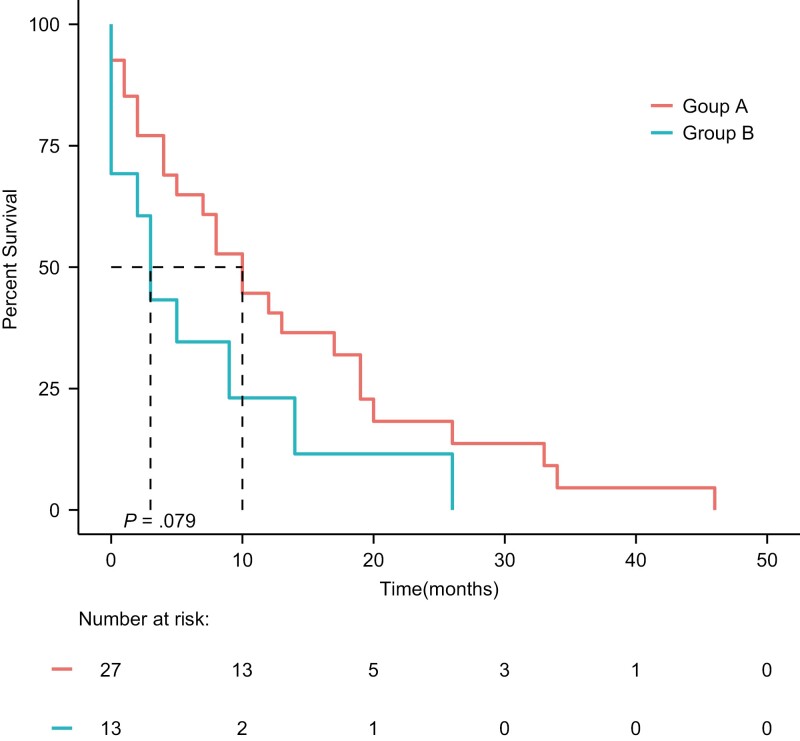
Time-to-next-treatment (TNT) of anaplastic lymphoma kinase (ALK)-positive patients according to the treatment given immediately following a second ALKi; further ALKi (group A) or chemotherapy (group B); third-line cohort (*n* = 40). Group A—median TNT 10 months (95% CI: 5-19). Group B—median TNT 3 months (95% CI: 0-NR). *P* = .079.

The TNT from the onset of the third line of interest was 10 months for group A (95% CI: 5-19) and 3 months for group B (95% CI: 0-NR, *P* = .085; Figure 3). On univariate analysis, the factors significantly associated with longer TNT on third line of interest was treatment with definitive or stereotactic radiotherapy (*P* = .025 regarding one such treatment, nonsignificant regarding more than one such treatment) and the numbers of ALKi given (*P* = .02). However, none of these were significant on multivariate analysis (Table 4). Third-line treatment with ALKi versus chemotherapy was not associated with a statistically significant different TNT in univariate nor in multivariate analysis (Table 4). As for survival, the difference in TNT between groups A and B was not statistically significant nor by HR evaluation of the Kaplan–Meier curves nor by evaluation of chance of treatment switch at land-mark time points (data not shown).

**Table 4.** Univariate and multivariate analysis of time-to-next-treatment of ALK positive patients treated with 2 ALKi and beyond, from initiation of third line of interest (*n* = 40).

	Univariate analysis		Multivariate analysis	
Parameters	HR (CI 95%)	*P*-value	HR (CI 95%)	*P*-value
Female vs Male	0.79 (0.39-1.57)	.500	0.52 (0.21-1.3)	.146
Age (years)	1.02 (0.99-1.05)	.121	1.02 (0.98-1.0)	.313
First-line Chemotherapy vs ALKi	0.77 (0.39-1.55)	.468	0.72 (0.29-1.8)	.464
BM at diagnosis	0.48 (0.21-1.12)	.090	0.55 (0.18-1.70)	.288
Definitive XRT (*N* of courses)				
0	Reference			
**1**	**0.36 (0.14-0.88)**	**.025**	0.57 (0.17-1.90)	.371
≥2	0.50 (0.18-1.36)	.174	0.56 (0.17-1.90)	.347
Palliative XRT (*N* of courses)				
0	Reference			
1	1.20 (0.54-2.64)	.657		
≥2	1.71 (0.57-5.12)	.341		
Total *N* chemotherapy lines	1.21 (0.81-1.81)	.344		
**Total *N* ALKi lines**	**0.49 (0.27-0.90)**	**.020**	0.56 (0.28-1.1)	.113
Time from diagnosis of advanced disease to start of third-line Tx	1.00 (0.98-1.03)	.811		
Group A vs Group B	0.51 (0.25-1.08)	.081	0.78 (0.26-2.30)	.652

Statistically significant parameters are highlighted in bold.

Group A; further ALKi beyond 2 ALKi, group B; chemotherapy beyond 2 ALKi.

Abbreviations: ALK, anaplastic lymphoma kinase; ALKi, ALK inhibitor; BM, brain metastases; CI, confidence interval; HR, hazard ratio; Tx – treatment; XRT, radiotherapy.

## Discussion

We have assembled a large set of high-resolution real-life data of ALK-positive metastatic NSCLC patients. We report a mature survival (47% maturity) outcome of a strikingly long median OS of 52 months (95% CI: 32-65). This result is in accordance with less-mature reported survival of clinical trials such as the PROFILE 1014 (41% maturity)^7^ reporting non-reached (NR) median OS for crizotinib-treated patients (95%CI: 45.8 months to NR), as well as other real-world ALK-positive cohorts, such as Gibson et al, who reported OS of 48.5 months in a cohort of ALK-positive NSCLC patients treated with ALKi.^19^ The more recent ALEX study reported a median OS that was NR with alectinib (33.6% maturity) and 57.4 months with crizotinib (95% CI: 34.6-NR; 41.1% maturity).^20^ Considering the expected worse outcome of real-world patients compared with clinical-trial patients, the outcome of our cohort is within the anticipated range. The only parameters found to be associated with longer OS of the full cohort were age and the number of ALKi treatment lines given. However, the choice of treatment immediately following the failure of the second ALKi, which was the main question we aimed to answer, was not associated with survival in a statistically significant manner.

In this study, we focused on a cohort of patients who were treated with 2 lines of ALKi and an additional treatment line following the failure of the second ALKi. Within this “third-line cohort” we compared 2 well-balanced groups of patients, those receiving ALKi versus those receiving chemotherapy following the failure of the second ALKi treatment. We found ALKi treatment at this point to be associated with a nonsignificant trend for better OS compared with chemotherapy. Qualitatively similar results were found regarding OS whether quantified from diagnosis of advanced disease or from initiation of the third line of interest. TNT also demonstrated a nonsignificant trend to be longer for the patients treated with ALKi as the third line of interest. Interestingly, one of the few factors that were significantly associated with better survival among the third-line cohort was the administration of chemotherapy as the first treatment line, before any ALKi, although this analysis was based on a small cohort of 44 patients, and 14 patients in the third-line cohort. The number of ALKi treatment lines was associated with survival but only with OS when quantified from initiation of third line of interest. As mentioned, treatment with a higher number of ALKi was significantly associated with OS also in the full cohort. These results might have been the result of a selection bias; it is conceivable that patients who survive longer would have been exposed to a larger number of treatment lines. However, in support of our finding, the final analysis of the Profile 1014 study demonstrated the longest survival in patients receiving 2 ALKi treatment lines versus only one such line.^7^

The clinical trials assessing second-line ALKi following progression on crizotinib were not randomized to include chemotherapy arm, although demonstrated prolonged PFS compared with the known outcomes with chemotherapy. Alectinib in crizotinib-refractory patients demonstrated PFS of 8.1 months,^21^ and brigatinib demonstrated PFS of 12.9 months in this patient population.^22^ As a very indirect comparison, first-line chemotherapy achieved a PFS of 7 months for ALK patients in the PROPHILE 1014 trial.^7^ Following the aforementioned studies, it has become standard of care to use advanced generation ALKi in the second line, even when second-generation agent was used in the first line, although brigatinib and alectinib were only prospectively assessed after first-generation ALKi, and the only ALKi assessed prospectively in the second and third line after second-generation ALKi was lorlatinib.^14^ Of note, some of the patients of group B in our study, treated with chemotherapy as a third-line therapy, were treated with additional line/s of ALKi afterwards, potentially masking the benefit of using a third ALKi.

Chemotherapy is clearly not the preferred option for first-line therapy in ALK-rearranged patients nowadays, as crizotinib demonstrated longer PFS over it,^6^ and a trend for longer OS,^7^ and the second-generation agents alectinib and brigatinib demonstrated superior PFS over crizotinib. An unplanned analysis of PROFILE 1014 using a statistical method to correct for crossover did demonstrate better OS for the crizotinib arm.^7^ In addition, metanalysis of prospective randomized trials of ALKi versus chemotherapy revealed PFS benefit using ALKi in the first line compared with chemotherapy, although no significant OS benefit was demonstrated.^23^ Further analysis suggests that the use of ALKi as the second line following first-line chemotherapy does not negatively impact survival in a significant manner.^23^ Interestingly, regarding the treatment of an analogous group of patients, namely EGFR-positive patients, recent studies suggest that combined chemotherapy and EGFR inhibitors as a first-line treatment can prolong PFS and OS over EGFR inhibitors alone.^24,25^ These results potentially are generalizable, implying that chemotherapy may play an important role in metastatic NSCLC with targetable driver mutation. Interestingly, among the patients receiving first-line chemotherapy in our study, in 61% of the cases, the switch to ALKi was done before disease progression. Because these patients’ diseases did not progress on chemotherapy, they potentially can be regarded as having received a treatment equivalent to first-line ALKi and chemotherapy. To our knowledge, there are no similar studies combining ALKi and chemotherapy, although this can be a reasonable treatment option for symptomatic or poor-prognosis patients, as ALK-positive patients can have a good response to platinum-based chemotherapy, and specifically to pemetrexed.^26^ Our real-world results puts forward the hypothesis that chemotherapy should be studied in combination with first-line ALKi.

We have found that a single course of definitive radiotherapy is associated with increased TNT on third-line therapy; this association was significant only on univariate analysis. It has become a common practice to treat isolated progression occurring on targeted therapy with local approaches, mainly radiotherapy. Our results do not negate further use of this treatment strategy, although care should be exercised considering the lack of OS benefit of XRT. A potential consideration in support of the use of definitive XRT for ALK-positive patients is Wang’s SINDAS trial, which demonstrated increased OS of metastatic NSCLC patients with EGFR mutation treated with aggressive radiotherapy in addition to EGFR TKI.^27^ Further studies of this issue are required.

Our study is limited by the small number of patients treated with 2 ALKi and beyond and by the retrospective nature of the trial. However, the data were collected by detailed chart analyses, compiling one of the largest datasets of ALK-positive patients that incorporates data of multiple treatment lines. Importantly, our data set includes information on additional therapies such as chemotherapy and radiotherapy and a long follow-up period, enabling an analysis of the impact of various interventions along the course of the disease. Another limitation of our study is the analysis of all ALKi as a group, without focusing on the sequence of the specific inhibitors. This was a choice made aiming to increase the power of our analysis, allowing us to point at a potentially important role of first-line chemotherapy, but not allowing us to suggest optimal ALKi sequence. Larger cohorts are required to study various ALKi sequences. It should be noted that the large majority of patients in our third-line cohort received crizotinib as the first-line treatment, thus limiting the interpretation of our results regarding patients initiating treatment with advanced generation ALKi. In addition, only 2 patients in cohort A of the third-line group were treated with lorlatinib, the only ALKi to date, which was validated prospectively and is indicated for third line in patients with ALK-positive NSCLC.^14^ Both of these treatment choices are expected considering the timeframe of this study, mostly during a period when crizotinib was the only first-line ALKi approved and lorlatinib was not available. To conclude, in this retrospective multi-institutional cohort of ALK-positive metastatic NSCLC, the choice of treatment in the third-line setting, whether a third ALKi or chemotherapy, did not impact survival in a significant manner. The number of ALKi lines administered was associated with increased survival. The use of definitive radiotherapy prolonged time-to-next-treatment of the third line of interest. Considering the limitations of real-world studies, our data can assist in choosing the optimal treatment option for ALK-positive patients in advanced treatment lines.

## Supplementary Material

oyab005_suppl_Supplementary_Figure

oyab005_suppl_Supplementary_Table

## Data Availability

The data underlying this article will be shared on reasonable request to the corresponding author.
