# Supplementary material for: ALK Inhibitors or Chemotherapy for Third Line in ALK-positive NSCLC? Real-world Data
Source: Oncologist. 2022 Jan 28;27(1):e76–84. doi: 10.1093/oncolo/oyab005 (PMC8842297; doi:10.1093/oncolo/oyab005)

The Oncologist
The official journal of the Society for Translational Oncology

Supplemental Figure for:
Alk inhibitors or chemotherapy for third-line ALK positive NSCLC? real world data
Jair Bar et al.

Supplementary Fig. S1. Overall survival of ALK-positive patients from diagnosis of advanced disease, according to the third-line of interest (i.e. treatment given immediately following a second ALKi course; further ALKi - group A, or chemotherapy- group B); third-line cohort (n=40). Group A - median OS 65 months (95% CI: 32-NR). Group B - median OS 55 months (95% CI: 46-NR). P = 0.12.

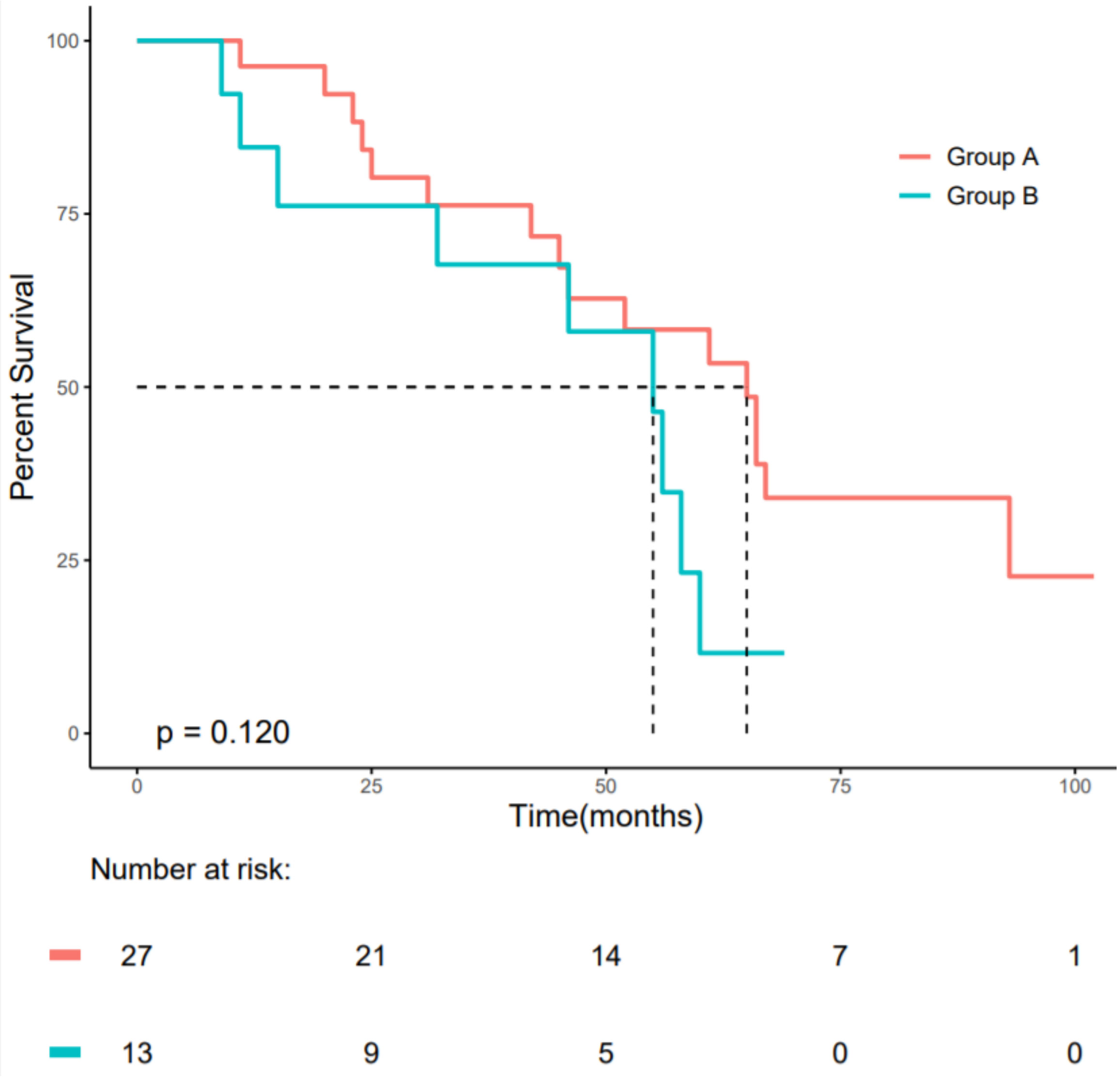

Supplement: oyab005_suppl_Supplementary_Figure [file oyab005_suppl_supplementary_figure.pdf]
