# Supplementary material for: ALK Inhibitors or Chemotherapy for Third Line in ALK-positive NSCLC? Real-world Data
Source: Oncologist. 2022 Jan 28;27(1):e76–84. doi: 10.1093/oncolo/oyab005 (PMC8842297; doi:10.1093/oncolo/oyab005)
Supplement: oyab005_suppl_Supplementary_Table [file oyab005_suppl_supplementary_table.pdf]

The Oncologist
The official journal of the Society for Translational Oncology

Supplemental Table for:
Alk inhibitors or chemotherapy for third-line ALK positive NSCLC? real world data
Jair Bar et al.

Supplementary Table S1

Cox proportional-hazards model *o*f univariate and multivariate analysis of OS from diagnosis of advanced disease for ALK-positive patients treated beyond two ALKi; third-line cohort (n=40). Statistically significant parameters are highlighted in bold.

	**Univariate analysis**		**Multivariate analysis**	
Parameters	HR (CI 95%)	P-value	HR (CI 95%)	P-value
Female vs. Male	1.13 (0.50-2.53)	0.762	0.73(0.25-2.12)	0.568
Age	1.01 (0.98-1.04)	0.444	1.01(0.98-1.05)	0.406
First-line Chemotherapy vs. ALKi	**0.25 (0.10-0.67)**	**0.006**	**0.17 (0.06-0.53)**	**0.002**
BM at diagnosis	0.81 (0.32-2.04)	0.661		
Definitive XRT (N of courses)				
0	Reference			
1	0.45 (0.18-1.15)	0.097	0.33(0.09-1.21)	0.094
≥2	0.57 (0.19-1.67)	0.306	0.63(0.14-2.78)	0.545
Palliative XRT (N of courses)				
0	Reference			
1	0.61 (0.23-1.60)	0.317		
≥2	1.43 (0.47-4.33)	0.524		
Total N	1.04 (0.58-1.85)	0.891		

chemotherapy lines				
Total N ALKi lines	0.54(0.28-1.05)	0.072	0.76(0.37-1.53)	0.437
Group A vs. Group B	0.52 (0.22-1.21)	0.129	0.87(0.29-2.59)	0.798

Group A; further ALKi beyond two ALKi, group B; chemotherapy beyond two ALKi. ALK- anaplastic lymphoma kinase; ALKi - ALK inhibitor; XRT - radiotherapy; BM - brain metastases.
